# Dominance reversals and the maintenance of genetic variation for fitness

**DOI:** 10.1371/journal.pbio.3000118

**Published:** 2019-01-29

**Authors:** Tim Connallon, Stephen F. Chenoweth

**Affiliations:** 1 School of Biological Sciences and Centre for Geometric Biology, Monash University, Clayton, Australia; 2 School of Biological Sciences, University of Queensland, St. Lucia, Australia; 3 Swedish Collegium for Advanced Study, Uppsala, Sweden

## Abstract

Antagonistic selection between different fitness components (e.g., survival versus fertility) or different types of individuals in a population (e.g., females versus males) can potentially maintain genetic diversity and thereby account for the high levels of fitness variation observed in natural populations. However, the degree to which antagonistic selection can maintain genetic variation critically depends on the dominance relations between antagonistically selected alleles in diploid individuals. Conditions for stable polymorphism of antagonistically selected alleles are narrow, particularly when selection is weak, unless the alleles exhibit “dominance reversals”—in which each allele is partially or completely dominant in selective contexts in which it is favored and recessive in contexts in which it is harmful. Although theory predicts that dominance reversals should emerge under biologically plausible conditions, evidence for dominance reversals is sparse. In this primer, we review theoretical arguments and data supporting a role for dominance reversals in the maintenance of genetic variation. We then highlight an illuminating new study by Grieshop and Arnqvist, which reports a genome-wide signal of dominance reversals between male and female fitness in seed beetles.

## Abstract 2

Dominance reversals between alleles can promote the maintenance of genetic variation, but direct experimental evidence for such effects is sparse. This Primer explores theories of dominance and evidence for dominance reversals, and highlights a new study reporting a genome-wide signal of dominance reversals for fitness in seed beetles.

## Introduction

Evolution by natural selection requires heritable variation for traits affecting fitness. Although most traits exhibit heritable variation [1], the high levels of genetic variation commonly reported for fitness-related traits represent a paradox [2–3]. Put simply, if natural selection typically decreases genetic variation by fixing beneficial genetic variants and removing harmful ones, then why is genetic variation so pervasive? And what evolutionary forces might maintain it?

Two evolutionary hypotheses are traditionally invoked to explain the maintenance of genetic variation for fitness and its underlying components of survival, fertility, fecundity, and mating success [4–5]. According to the mutation-selection balance hypothesis, variation is maintained at an equilibrium between recurrent mutation, which generates a steady stream of new, deleterious genetic variation, and natural selection which removes it. The balancing selection hypothesis, in contrast, proposes that genetic variation is maintained solely by natural selection. Balancing selection can take a variety of specific forms, including heterozygote advantage [6], negative frequency-dependent selection [7], and some forms of antagonistic selection between different environments (or “niches” [8]), sexes [9], or fitness components (e.g., survival versus fecundity [10]). It should be emphasized that antagonistic selection can result in either the maintenance of genetic variation or its loss and is not a sufficient condition for balancing selection [11].

The vast majority of mutations are harmful, so there is no question that mutation-selection balance contributes substantially to the maintenance of genetic variation. But is mutation-selection balance sufficient to explain the genetic variation observed in fitness-related traits? The answer appears to be no, at least in cases in which suitable data are available. For example, variation in some traits is affected by common alleles with large phenotypic effects, which is incompatible with the mutation-selection balance hypothesis (e.g., [12–13]). Population genomic studies have also identified a small, but expanding, list of genes and genomic regions that bear statistical signals of long-term balancing selection (e.g., [14–15]). Finally, research on *Drosophila* populations suggests that genetic variation for survival, fecundity, and mating success is too high to be explained by mutation-selection balance alone; the “excess” genetic variation for these traits is presumably due to balancing selection of some kind [16–18].

Despite evidence for balancing selection, doubts persist about its broader role in maintaining fitness variation. These doubts reflect the current deficit of clear-cut examples of balanced polymorphisms [19] and unanswered questions about the potential for specific mechanisms of balancing selection to account for broad-scale patterns of variation in fitness traits (e.g., [16]). Such doubts feature prominently in debates about the effect of sexually antagonistic (SA) selection on the maintenance of genetic variation. SA selection—which arises when traits that are favorable in one sex are disfavored in the other—is a prominent feature of many plant and animal populations ([20], but see [21]) and potentially generates balancing selection at SA genes (i.e., genes with alleles that benefit one sex at a cost to the other [22]). But like other scenarios of antagonistic selection, SA selection is not a sufficient condition for balancing selection [11], and the empirical database of known SA genes is presently far too small to determine the extent to which SA genetic variation is maintained by balancing selection as opposed to recurrent mutation (reviewed by [23–24]; see [25]).

In diploids, the evolutionary potential for SA selection to maintain variation hinges upon the dominance relations between SA alleles. Population genetics theory predicts that balancing selection is particularly likely when SA alleles exhibit “dominance reversals” between the sexes (e.g., female-beneficial alleles are dominant within females but recessive in males and vice versa for male-beneficial alleles [9,11]). Dominance reversals expand the scope for balancing selection at SA genes by generating a net heterozygote advantage at such loci. Although dominance reversals are predicted to arise under biologically reasonable conditions [26–30], empirical evidence for them is rare, though few studies systematically test for them. In this primer, we review theory and data on dominance reversals and their implications for balancing selection. We then highlight an exciting new study by Grieshop and Arnqvist [31], which reports evidence for extensive sex differences in dominance for fitness in a seed beetle population exhibiting high levels of SA genetic variation. The study takes a creative approach to infer sex differences in dominance and provides new support for dominance reversals as important population genetic mechanisms for maintaining genetic variation for fitness.

### Dominance reversals and balancing selection

Antagonistic selection—which generates genetic trade-offs between different contexts of selection or fitness—has long been suspected to contribute to the maintenance of genetic variation, yet this suggestion has been met with some debate [11]. On the one hand, fitness trade-offs are thought to be common because they arise naturally within even the simplest contexts of environmental variability [32], pleiotropy between traits or life stages [33], and sex differences in selection [34]. On the other hand, polymorphisms exhibiting trade-offs are rarely expected to be stably maintained, particularly when selection is weak, unless the alleles affecting the trade-off exhibit dominance reversals [11].

SA selection represents a special case of the broader debate (e.g., [10,11,27,35]), and its example clarifies the argument (Table 1; see [9]). Consider the following case of a single SA gene with two alleles—a female-beneficial allele (*A*_*f*_) and a male-beneficial allele (*A*_*m*_). In the model, *s*_*f*_ and *s*_*m*_ represent the costs to females and males, respectively, of being homozygous for the “wrong” allele (e.g., *s*_*f*_ is the cost to females of inheriting an *A*_*m*_ allele from both parents). The cost of being heterozygous for the gene depends on the dominance relations between the alleles, which can potentially differ between the sexes (see Table 1, in which *h*_*f*_ is the dominance coefficient of the *A*_*m*_ allele in females and *h*_*m*_ is the dominance coefficient of the *A*_*f*_ allele in males; therefore, *h*_*f*_ and *h*_*m*_ each refer to the dominance of the “wrong” allele in each sex). In this model, *h*_*f*_, *h*_*m*_, *s*_*f*_, and *s*_*m*_ are assumed to be constant, generations do not overlap, and fertilizations are random with respect to the genotypes of breeding adults.

**Table 1:** Sex-specific fitness at a diploid gene with SA alleles^1^.

Genotype	*A*_*f*_*A*_*f*_	*A*_*f*_*A*_*m*_	*A*_*m*_*A*_*m*_
**Female fitness**	*W*	*W*(1 − *s*_*f*_*h*_*f*_)	*W*(1 − *s*_*f*_)
**Male fitness**	*V*(1 − *s*_*m*_)	*V*(1 − *s*_*m*_*h*_*m*_)	*V*
**Mean relative fitness**^**2**^	1 − *s*_*m*_/2	1 − (*s*_*f*_*h*_*f*_ + *s*_*m*_*h*_*m*_)/2	1 − *s*_*f*_/2

^1^*W* and *V* scale the relative fitness values of the three genotypes to absolute fitness. Remaining parameters are assumed to fall with the biological range: 0 < *s*_*f*_, *s*_*m*_ < 1; 0 < *h*_*f*_, *h*_*m*_ < 1.

^2^Relative fitness for each sex is scaled against the fitness of the best genotype in that sex (e.g., for females: 1, 1 − *s*_*f*_*h*_*f*_, and 1 − *s*_*f*_ for genotypes *A*_*f*_*A*_*f*_, *A*_*f*_*A*_*m*_, and *A*_*m*_*A*_*m*_, respectively). The mean relative fitness of each genotype is the average of the male and female relative fitnesses. Net overdominance occurs when the mean relative fitness of the *A*_*f*_*A*_*m*_ genotype is higher than the mean relative fitness of both homozygous genotypes.

**Abbreviations:** *A*_*f*_, female-beneficial allele; *A*_*m*_, male-beneficial allele; *h*_*f*_, dominance coefficient of the *A*_*m*_ allele in females; *h*_*m*_, dominance coefficient of the *A*_*f*_ allele in males; SA, sexually antagonistic; *s*_*f*_, the cost to females of being homozygous for the *A*_*m*_ allele; *s*_*m*_, the cost to males of being homozygous for the *A*_*f*_ allele.

Kidwell and colleagues [9] showed that balancing selection at the gene requires the following condition to be true:

$$\frac{s_{m}h_{m}}{1-h_{f}+s_{m}h_{m}}<s_{f}<\frac{s_{m}(1-h_{m})}{h_{f}(1-s_{m})}.$$ (1)

**Fig 1:**
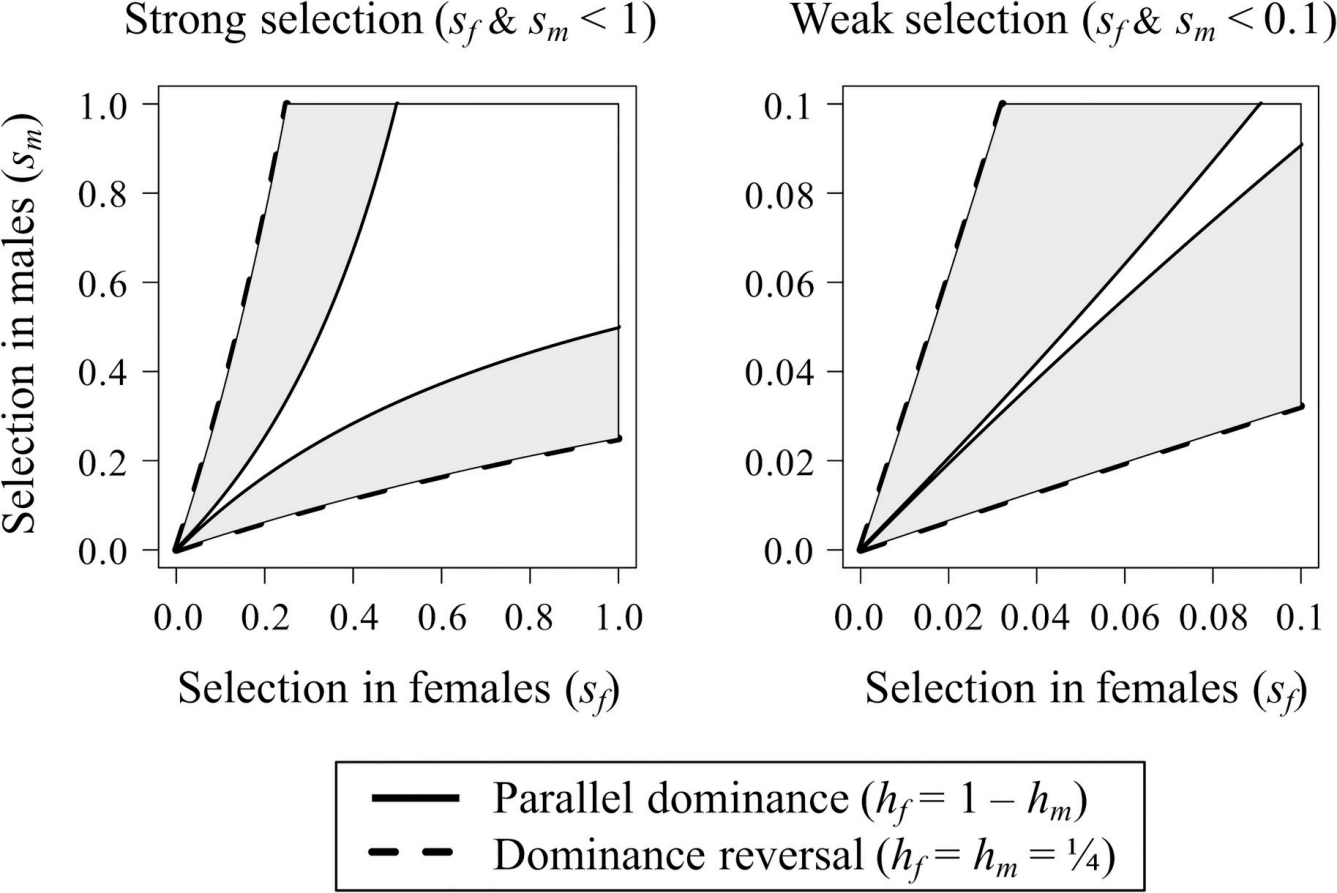
Dominance reversals promote balancing selection at an SA gene. Left panel: strong selection. Right panel: weak selection. The regions between the solid black curves show the conditions for balancing selection under parallel dominance (*h*_*f*_ = 1 − *h*_*m*_). The regions between the dashed lines show the conditions for balancing selection under a partial dominance reversal, where *h*_*f*_ = *h*_*m*_ = ¼. The grey shaded regions show the expanded parameter space for balancing selection caused by the dominance reversal. This expanded parameter space due to dominance reversal is particularly pronounced when selection is modest to weak (right panel). Stronger dominance reversals (*h*_*f*_ and *h*_*m*_ < ¼) further expand the conditions for balancing selection. Theoretical curves are based on Eq 1, and the figure is based on Figs 1 and 3 of Kidwell and colleagues [9]. *A*_*f*_, female-beneficial allele; *A*_*m*_, male-beneficial allele; *h*_*f*_, dominance coefficient of the *A*_*m*_ allele in females; *h*_*m*_, dominance coefficient of the *A*_*f*_ allele in males; SA, sexually antagonistic; *s*_*f*_, the cost to females of being homozygous for the *A*_*m*_ allele; *s*_*m*_, the cost to males of being homozygous for the *A*_*f*_ allele.

When *h*_*f*_ = 1 − *h*_*m*_, each allele exhibits the same pattern of dominance in each sex (e.g., the *A*_*f*_ allele is dominant to *A*_*m*_ in both sexes when 0 < *h*_*f*_ < ½; *A*_*m*_ is dominant to *A*_*f*_ when ½ < *h*_*f*_ < 1). This scenario is known as “parallel dominance” [10–11]. Under parallel dominance, sex asymmetries in the strength of selection (i.e., differences between *s*_*f*_ and *s*_*m*_) typically lead to directional selection and fixation of one of the two alleles. It is only when selection is very strong (i.e., *s*_*f*_ and *s*_*m*_ >> 0) or near perfectly balanced between the sexes (*s*_*f*_≈*s*_*m*_) that balancing selection is likely (Fig 1). Otherwise, SA selection removes genetic variation at the gene. Under parallel dominance, mean relative fitness of the heterozygous genotype is always intermediate between the fitnesses of the two homozygous genotypes (provided 0 < *h*_*f*_ < 1); consequently, there is no net heterozygote advantage at the locus, even in cases in which polymorphism is maintained (see Table 1).

Beneficial reversals of dominance result in the deleterious allele of a given sex becoming partially masked within that sex (i.e., *h*_*f*_ and *h*_*m*_ < ½, in which the *A*_*m*_ allele is recessive in females and the *A*_*f*_ allele is recessive in males). Such dominance reversals favor polymorphism under a much broader range of conditions than in the parallel dominance scenario (Fig 1), and in the extreme case of a complete reversal of dominance (*h*_*f*_ and *h*_*m*_ = 0), balancing selection becomes inevitable [9] (for recent theory that builds upon this framework, see [27,36]). Dominance reversals can lead to a net heterozygote advantage for mean relative fitness (see Table 1), which expands conditions for balancing selection under SA selection.

In contexts in which the condition for balancing selection is not met (i.e., inequality [Eq 1] is false), SA alleles may still contribute disproportionately to fitness variance compared to other classes of mutations [25]. This effect arises because SA alleles that are ultimately destined for fixation or loss will, nevertheless, have long persistence times in the population relative to unconditionally beneficial or harmful mutations; long persistence times elevate the contributions of transient SA polymorphisms to standing genetic variation for fitness. In cases in which polymorphisms are not stably maintained, dominance reversals extend the persistence times of SA alleles.

### Evolutionary theories of dominance and dominance reversals

At first glance, one might expect dominance reversals to be uncommon. Why should beneficial effects of SA alleles be dominant, whereas their fitness costs remain recessive? Such a scenario is undoubtedly fortuitous for heterozygous individuals, who secure most of the fitness gains associated with SA alleles without incurring much of their costs. On the other hand, such fortuitousness seems too good to be true without a clear biological mechanism to explain why dominance reversals might arise in the first place. As we review below, classical theories of genetic dominance provide such a mechanism. Indeed, these theories predict that dominance reversals should be particularly common among mutations that trade-off between environments, sexes, or fitness components.

**Fig 2:**
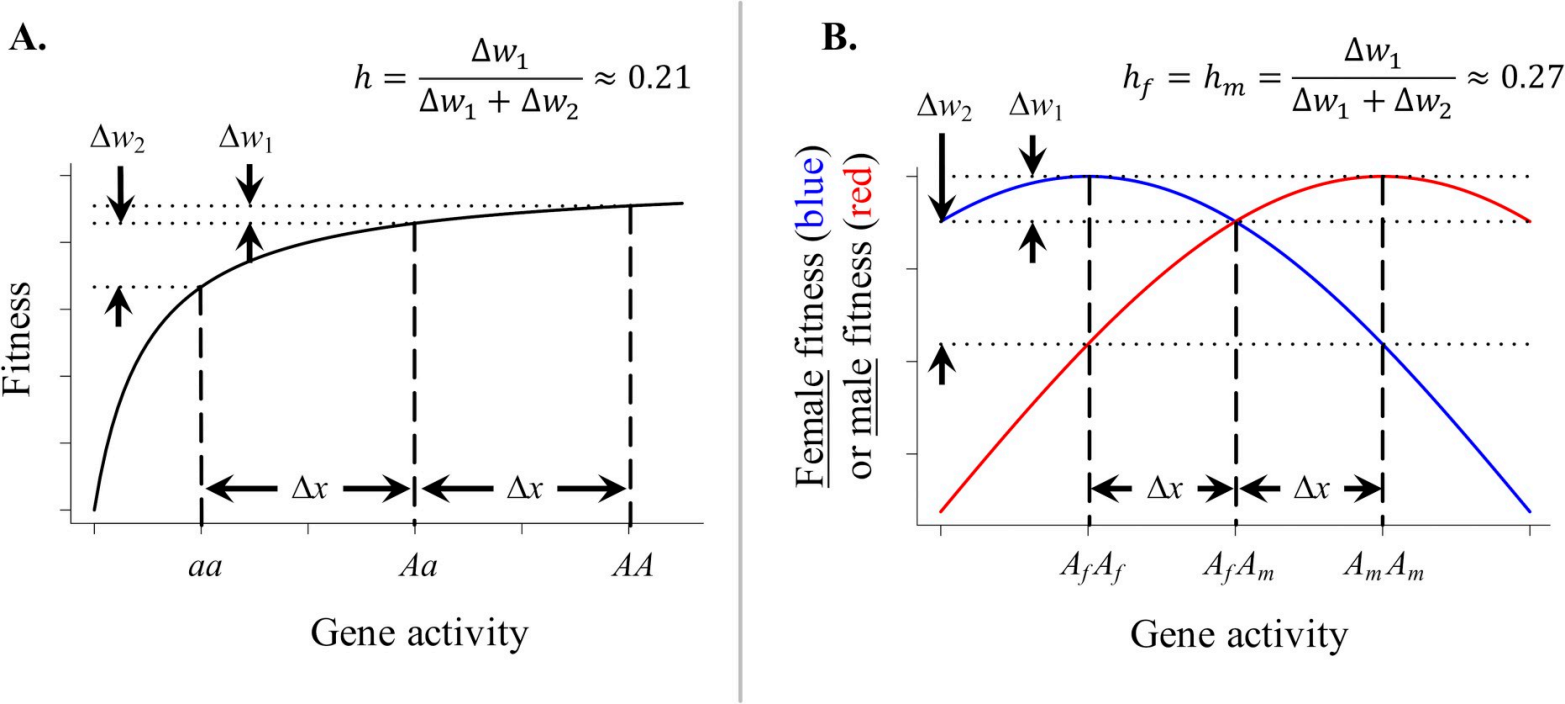
Dominance emerges from concave fitness surfaces. (A) Wright’s theory of dominance (based on Fig 7 from Wright [41] and Fig 1 from Otto and Bourguet [38]). A concave relation between gene activity and fitness causes deleterious mutations to be partially recessive to beneficial ones. In the example, a beneficial allele, *A*, and deleterious allele, *a*, have additive effects on gene activity (i.e., alleles alter gene transcription or function by amount Δ*x*). The diminishing-return relation between fitness and gene activity results in partial recessivity of *a* with respect to fitness (*h* < ½). (B) Dominance reversal at an SA gene (based on Fig 1 from Gillespie and Langley [44] and Fig 2 from Fry [27]). The fitness surfaces for females and males (in blue and red, respectively) are each concave but have different optima. The SA alleles have additive effects on gene activity (i.e., by Δ*x*); the concave mapping of fitness on gene activity causes the deleterious variant for each sex to be partially recessive (*h*_*f*_ and *h*_*m*_ < ½), so that *A*_*f*_ is partially recessive in males and *A*_*m*_ is partially recessive in females. *A*_*m*_, male-beneficial allele; *h*_*f*_, dominance coefficient of the *A*_*m*_ allele in females; *h*_*m*_, dominance coefficient of the *A*_*f*_ allele in males; SA, sexually antagonistic.

The earliest debate over the evolutionary causes of dominance pitted R. A. Fisher against Sewall Wright, initiating a conceptual split within the field of evolutionary biology and resulting in much personal acrimony between the two theoreticians (see [37–38]). The debate sought to explain the widespread observation that harmful mutations are typically recessive or partially recessive with respect to their fitness costs. Fisher [39] argued that natural selection should favor the evolution of recessivity—that genetic systems should evolve to strongly mask the expression of harmful mutations. Wright [40] countered by showing that the strength of selection to modify dominance of a deleterious allele would be on the same order of magnitude as the mutation rate and thus too weak to be of evolutionary consequence. Wright posited, instead, that the observed dominance relations between deleterious and beneficial alleles could arise if fitness followed a diminishing-return function of gene activity (e.g., of enzyme catalytic activity [40–41]). The diminishing-return relation between genotype and fitness causes deleterious alleles to be partially recessive with respect to fitness (i.e., the *a* allele has a dominance coefficient of *h* < ½; see Fig 2A), as is widely observed among harmful mutations [42].

The key to Wright’s theory of dominance is the concave relation between genotype and fitness, which converts additive effects of alleles at the molecular level (e.g., enzyme activity in Wright’s model) to nonadditive effects for fitness. The beauty of Wright’s theory is that it applies broadly to other scenarios of trait variation. For example, when alleles of a gene have additive effects on a quantitative trait, a concave relation between trait expression and fitness will similarly convert additivity at the trait level to nonadditivity for fitness ([42–43]; see Fig 2, in which alleles have additive effects on the “gene activity” trait, and nonadditive fitness effects by way of the nonlinear relation between gene activity and fitness). When fitness effects of alleles are context dependent, as is the case for SA alleles (e.g., Table 1; Fig 2B), then dominance reversals arise naturally, as a consequence of concave and multipeaked fitness surfaces ([26–27,29,44]; see Fig 2B). In this case, mutations that move the trait closer to the optimum in one fitness context are partially dominant within that context.

The theory outlined above shows that dominance reversals may be intrinsic properties of SA alleles, yet Fisher’s theory of dominance may also contribute to, and possibly reinforce, the evolution of dominance reversals at SA genes [30]. Wright’s original argument against Fisher’s theory is valid for unconditionally deleterious mutations, which generate weak selection for dominance modification because they are rare. SA alleles need not be rare, and the evolution of dominance, including elaboration of dominance reversals, becomes plausible in cases in which heterozygosity is high [30,38].

### Empirical patterns of dominance for fitness and its components

What are the typical patterns of dominance for SA alleles? Although the theory outlined above predicts that beneficial effects should be partially dominant and deleterious effects partially recessive, these predictions are difficult to directly confirm. Our ignorance reflects two logistical challenges. The first is the great difficulty of identifying and characterizing SA genes [23–24]. The second is the difficulty of evaluating dominance among beneficial mutations in general [45], including conditionally beneficial mutations that are favorable in some contexts and deleterious in others (e.g., SA mutations).

Most of what is known about the dominance of fitness-altering mutations applies to deleterious alleles—by far the most abundant class of mutations. Deleterious alleles exhibit a wide range of dominance coefficients, though the typical deleterious mutation is partially recessive (roughly *h* ~ ¼, on average; reviewed in [42]), with sterile and lethal alleles more strongly recessive [46]. These patterns are consistent with the theoretical predictions outlined in the preceding section (see [42,47]). In contrast, we know surprisingly little about the dominance of other classes of mutations. Dominance is common among beneficial alleles that have contributed to adaptive divergence in outcrossing populations [48–49], though these variants are expected to show stronger dominance than new beneficial mutations [50].

Even less is known about the dominance of mutations involved in fitness trade-offs—a class of conditionally beneficial alleles. Even so, the existence of dominance reversals for fitness-related traits has been known for at least 60 years. For example, F1 crosses between mutant strains of *Arabidopsis thaliana* grown at different day lengths showed alternating dominance effects for flowering time, a key fitness trait in this species [51]. More recently, a genome-wide assay of gene expression traits in *Drosophila* revealed apparent dominance reversals between high and low temperatures for 1,384 genes [52], though the fitness consequences of the transcriptional variants are unclear. Several other examples of dominance reversals are known, all involving major-effect loci. For example, sickle-cell alleles of the *HbA* gene in humans are dominant with respect to malaria resistance but recessive with respect to anemia (discussed in [10]). Johnston and colleagues [12] showed that one allele of the *RXFP2* gene in Soay sheep has a dominant beneficial effect on male mating success (through increased horn volume) and a recessive deleterious effect on male survival. Recently, Barson and colleagues [13] reported a dominance reversal between the sexes at the *VGLL3* gene of Atlantic salmon; the late-maturation allele of *VGLL3* is partially dominant in females, in which it is thought to be beneficial, and recessive in males, in which it is thought to be deleterious.

These case studies confirm that dominance reversals can and do occur between different contexts of natural selection. Nevertheless, it remains unclear whether dominance reversals for antagonistically selected alleles are common across the genome, and what fraction of genetic variance for fitness they might account for. Given the large number of variable loci that affect organismal fitness, these questions are difficult to address on a gene-by-gene basis. Instead, they are more suited to novel applications of the biometric tools of classical quantitative genetics, as in the study by Grieshop and Arnqvist [31].

### Dominance reversals and SA variation in seed beetles

Seed beetles have emerged as a leading system for the study of SA selection and SA genetic variation ([31] and references therein). The system is exceptional for its experimental tractability, including the feasibility of large-scale breeding experiments and empirical measurements of fitness in laboratory environments that mimic those in which these beetle populations have evolved. Prior research on a population from Lomé (Togo, West Africa) documented a strong empirical signal of SA genetic variation.

**Fig 3:**
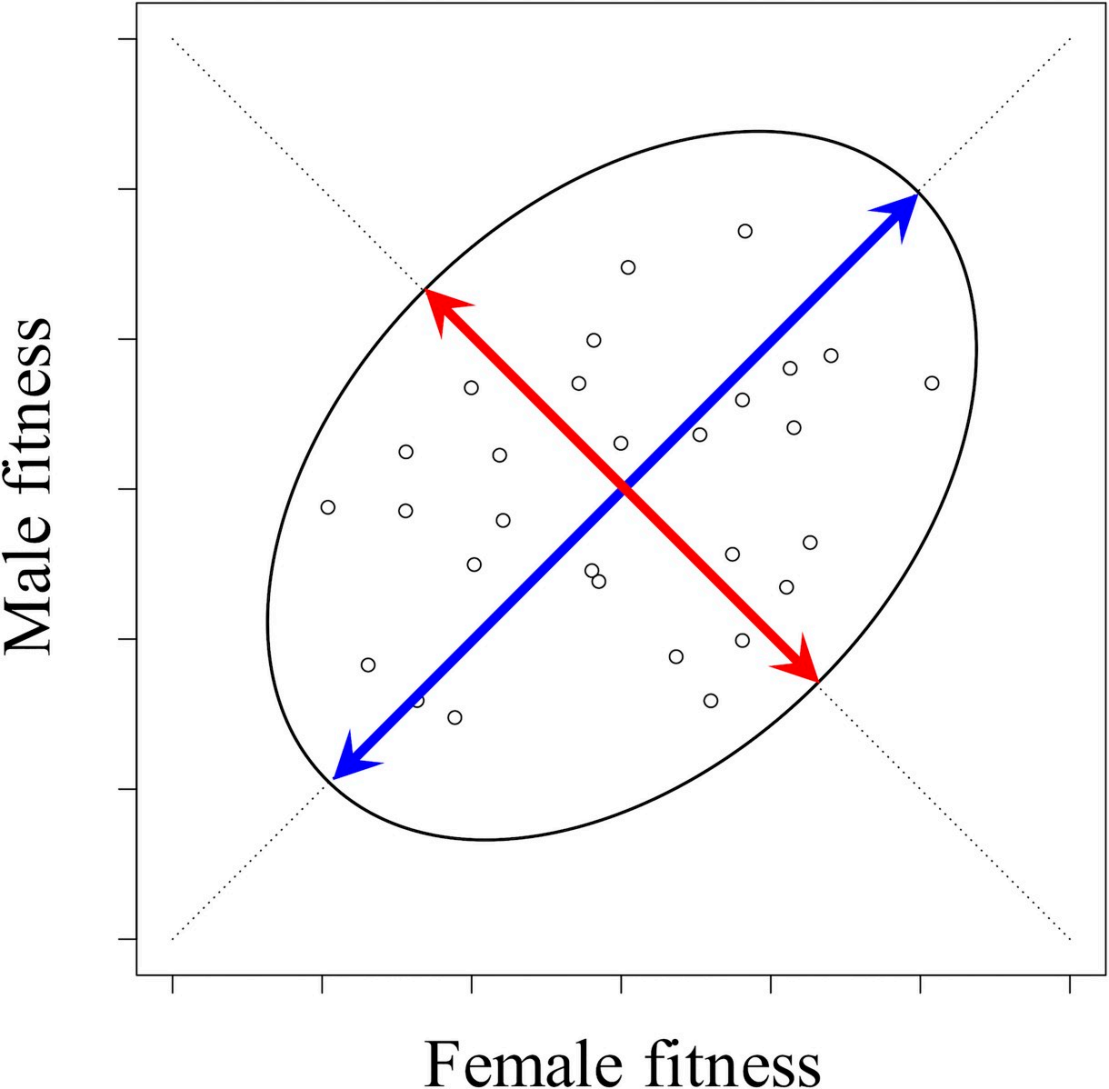
Axes of SA and sexually concordant fitness variation. The sexually concordant axis of genetic variation is marked in blue. The SA axis of genetic variation is marked in red. Circles show fitness estimates for a set of hypothetical experimental genotypes. SA, sexually antagonistic.

Using 16 inbred lines from the Lomé population, which capture a representative sample of genetic variation, Grieshop and Arnqvist [31] carried out a full diallel cross and assayed female and male lifetime fitness for each of 256 experimental genotypes. The crossing design allows for the genetic dissection of female and male fitness variation, while controlling for effects of maternal and paternal genetic transmission on offspring fitness. Their analysis focused on the genetic basis of variation along two major axes of sex-specific fitness: (1) a sexually concordant axis of variation, representing genotypic variation with similar effects on female and male fitness (Fig 3, in blue), and (2) an SA axis, representing SA variation (Fig 3, in red). Sexually concordant dominance variance, SA additive variance, and SA dominance variance were the primary components of overall fitness variation in the population—consistent with the hypothesis that balancing selection and dominance reversals contribute to the maintenance of fitness variation.

To test for sex differences in dominance among mutations that contribute to SA fitness variation, Grieshop and Arnqvist used an analytical method from classical quantitative genetics—applied separately to each sex—that ranks inbred lines by the dominance of causal alleles that each line carries. These line rankings are expected to be similar between the sexes when alleles contributing to SA fitness variance show similar dominance relations in each sex; dominance reversals should cause divergence of the line rankings between the sexes. Here, Grieshop and Arnqvist’s results could not be clearer: the rank order of dominance is reversed between the sexes, providing strong support for dominance reversals underlying the SA axis of fitness variation.

Grieshop and Arnqvist’s study establishes a compelling new link between dominance reversals and empirical patterns of sex-specific genetic variation for fitness—a link that has long been predicted by evolutionary theory yet has lacked the “smoking gun.” Not only does the study bolster the empirical case for dominance reversals in fitness variation, but its clever blend of classical breeding design and sophisticated quantitative genetic analysis should provide a useful guide for future work on the role of genetic trade-offs and balancing selection in the maintenance of genetic variation for fitness.

### Where to from here?

The theoretical requirements for dominance reversals are not restrictive, suggesting that antagonistically selected polymorphisms (even weakly selected ones) may often meet conditions for balancing selection. Indeed, even in the absence of nonadditive gene action underlying trait variation (as implied from genome-wide association studies [GWAS] for many human quantitative traits; see [53]), dominance reversals for fitness merely require that fitness surfaces are concave, so that beneficial mutational effects are partially dominant and deleterious effects are partially recessive [41–42]. Likewise, SA alleles—those that move one sex closer to its optimum and the other sex away—should show sex-specific dominance reversals provided the relation between trait expression and fitness is concave for each sex. The concavity requirement should be easy to meet for traits subject to stabilizing selection around an optimum, and empirical measures of fitness surfaces confirm that fitness is commonly a nonlinear function of morphological and transcriptional trait variation [54–56].

Grieshop and Arnqvist’s [31] study goes much further by identifying an approach to infer genome-scale patterns of dominance reversals for female and male fitness variation and, in the process, shows that sex-specific dominance reversals play important roles in maintaining fitness variation in seed beetles. The study validates evolutionary theories of dominance and dominance reversals, and their example suggests a fruitful avenue for future empirical progress: applying similar tests in other taxa and between other types of selective contexts may take us forward in understanding the extent to which antagonistic selection shapes genetic variation for fitness.

## Abbreviations

A_f_: female-beneficial allele
A_m_: male-beneficial allele
GWAS: genome-wide association studies
h_f_: dominance coefficient of the *A*_*m*_ allele in females
h_m_: dominance coefficient of the *A*_*f*_ allele in males
SA: sexually antagonistic
s_f_: the cost to females of being homozygous for the *A*_*m*_ allele
s_m_: the cost to males of being homozygous for the *A*_*f*_ allele
